# Obstetrical and Fertility Outcomes Following Transcatheter Pelvic Arterial Embolization for Postpartum Hemorrhage: A Cohort Follow-Up Study

**DOI:** 10.3390/life12060892

**Published:** 2022-06-15

**Authors:** Anda-Petronela Radan, Sophie Schneider, Jarmila A. Zdanowicz, Luigi Raio, Nando Mertineit, Johannes Thomas Heverhagen, Daniel V. Surbek

**Affiliations:** 1 Department of Obstetrics and Gynecology, University Hospital of Bern, 3010 Bern, Switzerland; sophie.schneider@insel.ch (S.S.); jarmila.zdanowicz@insel.ch (J.A.Z.); luigi.raio@insel.ch (L.R.); daniel.surbek@insel.ch (D.V.S.); 2 Department of Diagnostic, Interventional and Pediatric Radiology, University Hospital of Bern, 3010 Bern, Switzerland; nando.mertineit@gmail.com (N.M.); johannes.heverhagen@insel.ch (J.T.H.)

**Keywords:** embolization, fertility, postpartum hemorrhage

## Abstract

**Objectives:** Management of severe postpartum hemorrhage (PPH) includes transcatheter pelvic arterial embolization (TAE). Data regarding subsequent fertility and obstetrical outcomes is limited, as most fertility outcomes derive from TAE in uterine fibroma. The purpose of our study was to evaluate the long-term outcomes of patients undergoing TAE, particularly concerning subsequent fertility and following pregnancies. **Material and methods:** We included 28 patients who underwent TAE for PPH at our institution between 2009 and 2018 in a retrospective cohort study. Data were assessed by reviewing patients’ charts and by contacting the patients. **Results:** Ten patients had prophylactic balloon occlusion before cesarean section because of anticipated PPH, with planned hysterectomy by placenta increta/percreta. All these patients were excluded from the analysis regarding fertility. 16 (73%) patients reported having regular menstruation after TAE. In total, 11 women had no desire for subsequent pregnancy. Seven of the remaining 11 patients (63.6%) had a total of 13 spontaneous pregnancies, nine of these resulted in miscarriages. Four patients delivered a live baby (36.4%). Two of these (50%) had recurrent PPH and treatment was conservative. Of the patients with infertility (*n* = 4, 36.4%), two (18.1%) underwent assisted infertility treatment without success. **Conclusion:** Our study suggests that the fertility of patients undergoing TAE due to PPH is limited. In women who conceive, the risk for first trimester miscarriage as well as recurrent PPH seems to be increased. If this is a consequence of the underlying cause of PPH or the TAE remains unknown. Larger follow-up cohorts are needed. In the meantime, patients who desire pregnancy after TAE should be counseled accordingly.

## 1. Introduction

Postpartum hemorrhage (PPH) can be a life-threatening condition and represents one of the leading causes of maternal morbidity and mortality worldwide [1,2]. Despite modern measures of prevention, PPH incidence still reaches 5% of all deliveries, with a severe course in 1% of cases and higher mortality in underdeveloped countries [3,4]. Primary PPH occurs in the first 24 h after delivery, whereas secondary PPH can occur anytime within six weeks after birth. The most common cause of PPH is uterine atony. The strongest predictors for severe blood loss in the context of PPH are previous cesarean section (CS), prolonged labor, oxytocin administration, and emergency CS [5]. Due to its severity, prompt and adequate intervention is essential in managing PPH. Selective transcatheter arterial embolization (TAE) was introduced in 1979 as second-line therapy for severe PPH when conservative measures fail [1,6]. Compared to extensive emergency pelvic surgery in PPH, which often implies vessel ligation, compression sutures, or even hysterectomy; it is a minimally invasive procedure. For this reason, international guidelines recommend considering TAE prior to surgery, in cases where a specialized radiology center is accessible in a timely manner [7,8]. TAE has been proven an effective method, as hemostasis is reached in over 90% of the cases [9,10,11]. The American College of Obstetricians and Gynecologists included 2017 TAE in their recommendations for treatment of PPH, with the purpose of uterus preservation and potentially future fertility [12]. Furthermore, the International Federation of Gynecology and Obstetrics (FIGO) also states in its recommendations for 2022 for PPH management that ‘uterine artery embolization has become recognized as a relatively safe technique when preserving the patient’s fertility is a priority’ [13]. As TAE is not a routine intervention, no large follow-up series exist for patients who underwent the procedure, as opposed to fibroid embolization, where data are more extensive [14]. Counseling women prior to catheter embolization, particularly in terms of fertility preservation and outcomes in future pregnancies, can be challenging. Most studies report the preservation of unimpaired fertility after the procedure [9,10,15,16]. In addition, studies to date have reported either no menstrual disorders or a high resumption of normal menstruation after TAE [16,17,18,19]. In terms of future obstetrical outcomes, most papers report a higher risk for recurrent PPH [15,18,19]. Some report the occurrence of fetal growth restriction or abnormal placentation, whereas others describe no subsequent obstetrical complications after TAE [15,17,18,19,20,21]. The aim of this study was to assess and analyze obstetrical and fertility outcomes in women who underwent TAE for PPH in our primary care Obstetrical Center.

## 2. Materials and Methods 

We included in our retrospective cohort study all patients who underwent pelvic arterial embolization for PPH at our institution since 2009 to date, with a total of 28 patients. Data were collected by reviewing patients’ charts regarding medical procedures and by directly contacting the patients to assess long-term gynecological and fertility outcomes. Besides being contacted on the phone, each patient received a questionnaire regarding the desire for future conception and outcomes of subsequent pregnancies, when applicable. Furthermore, information regarding menstruation after TAE for PPH was assessed. One of the purposes of the questionnaire was to avoid bias in data collection. The local Ethics Committee approved the study (reference number 2016-00415). PPH was defined as a blood loss of more than 500 mL after vaginal delivery, respectively 1000 mL after cesarean section. The algorithm for managing PPH at our institution includes fluid management, administration of uterotonic drugs such as carbetocin (prophylactically), oxytocin and sulprostone, tranexamic acid, and, if required, fibrinogen, activated recombinant human factor seven (rhFVIIIa), red blood cell units (RBCUs), fresh frozen plasma (FFP) and platelets (PLT). Surgical interventions include management of genital tract lacerations, instrumental uterine revision, compression sutures, uterine tamponade with a catheter or surgical towels (with or without uterotonics), and arterial ligation. Although a further efficient alternative is represented by Celox^®^ uterine tamponade; this method has not been yet implemented as routine in our hospital and was not applied in the management of the patients in this study. Furthermore, management may vary depending on the local practice in case the delivery did not take place at our institution. TAE was performed by using permanent or resorbable embolic agents. In the case of prophylactic, preoperative therapy, a balloon occlusion catheter was used for temporary arterial blockage. Embolization was performed via femoral arterial access. The embolization sites are described in Table 1. Procedure for uterine artery embolization with embolic agents: Under sterile conditions and local anesthesia with 10 mL lidocaine, retrograde puncture of the common femoral artery and insertion of a 4–6 French sheath on both sides. Subsequent super selective probing using a single-curve catheter of the uterine artery on each side via the corresponding internal iliac artery. Closure with an embolic agent (Table 1). Procedure for prophylactic catheter occlusion: Under sterile conditions and local anesthesia with 10 mL lidocaine, retrograde puncture of the common femoral artery, and insertion of a 6 French sheath on both sides. Selective probing of the internal iliac artery on both sides. Insertion of the balloon catheter to the proximal part of the internal iliac artery on both sides.

## 3. Results

**Figure 1:**
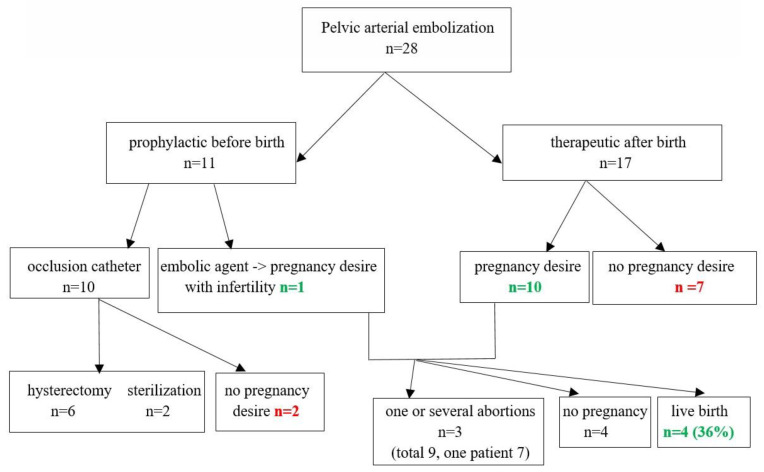
Study population.

Figure 1 summarizes our study cohort. 

In 18/28 patients (64%), delivery occurred in our center, whereas 10/28 patients (36%) were referred to our primary care center after giving birth in a non-tertiary hospital. Patient characteristics and obstetrical outcomes are summarized in Table 2. 

In 17 (61%) patients, pelvic arterial embolization was performed after failure of conservative medical treatment and hemodynamic stabilization of the patient, with therapeutic intention. In 11 (39%) patients, either balloon occlusion (*n* = 10) or TAE (*n* = 1) were performed prophylactically because of anticipated PPH before cesarean section or curettage by abnormally invasive placenta (AIP) (Figure 1). Six of these 11 patients required a concomitant hysterectomy, which was planned preoperatively, and in two women, sterilization was performed. Further, two patients had no desire for a subsequent pregnancy, thus in the ‘prophylactic group’, solely the patient receiving prophylactic TAE strived for pregnancy after the intervention. All patients treated by balloon occlusion, with or without uterus preservation, were excluded from the fertility analysis. In one patient, repeat TAE was performed: during CS and after surgery due to reoccurring severe hemorrhage. The main cause of PPH was uterus atony (40%), followed by abnormally invasive placenta or retained placenta and cervix/perineal lacerations. TAE was performed by using permanent or resorbable embolic agents (microspheres/particles, coils, or comminuted absorbable gelatin sponge). Prophylactic artery occlusion was performed by insertion of a balloon occlusion catheter in the desired pelvic artery and balloon inflation to temporarily block the perfusion. A single femoral arterial access approach was used in all cases. No case of extravasation was noted after performing final angiography and no immediate complications of vascular interventions occurred. In six of the patients with prophylactic balloon occlusion, blood loss was under 1000 mL. All further patients presented with PPH (*n* = 22) and required blood transfusions, on average 6.2 red blood cell units (RBCUs) being administered. 14 (57%) patients additionally required administration of fresh frozen plasma, and 8 (29%) women received platelet transfusions. Interventions for our patients are summarized in Table 3.

In multiple linear regression, none of the following factors correlated with the amount of blood loss in our cohort: maternal age, previous PPH, previous CS or curettage, presence of fibroids, and low-lying placenta (*p* > 0.05 for all co-variates). 

### Follow-Up Results

Sixteen (73%) of the patients who did not undergo hysterectomy reported having regular menstruation after TAE, two of them only after hysteroscopy was performed (either because of cervical stenosis or presence of Ashermann syndrome). In three (17%) patients, menstruation was either irregular or weak. One of the patients reported having lactational amenorrhea. In one patient, information regarding the resumption of menstruation was missing. After excluding all cases with prophylactic balloon occlusion, 18 cases remained in the cohort (17 therapeutic TAE and one prophylactic). Of these, 7 women had no desire for a subsequent pregnancy (Figure 1). Seven of the remaining 11 patients (63%) had a total of 13 spontaneous pregnancies. Nine of these pregnancies resulted in a miscarriage in the first trimester, seven of them in the same patient. Four (4/11, 36.4%) patients eventually delivered a live baby: one delivered vaginally after having delivered vaginally in the previous pregnancy (25%) and three underwent cesarean section (75%)—one elective CS and two repeat CS. Two patients (50%) had a recurrent postpartum hemorrhage, which was successfully treated by conservative measures. One patient suffered from recurrent placenta accreta and one pregnancy presented with vasa previa. In two patients, late preterm delivery was reported (Table 4). Of the patients with infertility (*n* = 4, 36.4%), two (18.1%) underwent assisted reproductive technology (ART) treatment without success. In logistic binary regression, total blood loss was not found to significantly influence the occurrence of infertility/sterility. The analysis of the embolization method as co-variate influencing the occurrence of infertility/sterility was not possible due to the limited number of cases.

## 4. Case Report

**Figure 2:**
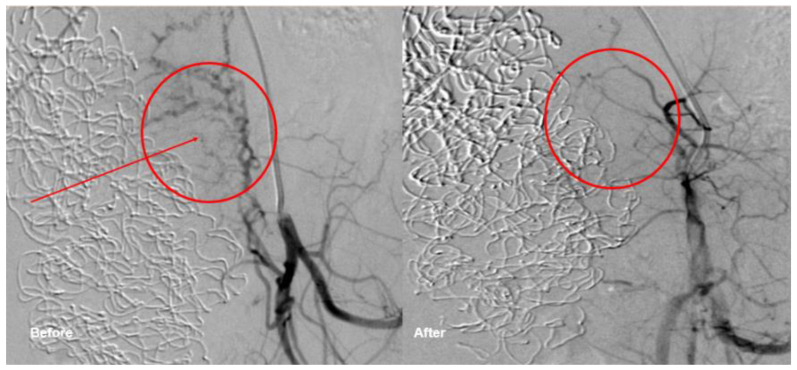
Transcatheter embolization at the left uterine vessels.

In the following, we present the case of an uncomplicated pregnancy after TAE. This 23-year-old primiparous woman with no pertinent medical history was admitted to a non-tertiary hospital at 40 weeks of gestation for rupture of membranes and beginning labor. In the second stage of labor, fetal bradycardia led to vacuum extraction of a healthy male newborn. Oxytocin (5 IU) was administered intravenously as a bolus; the placenta expulsion occurred ten minutes after delivery. Due to vaginal bleeding despite adequate uterine tonus, 1000 mcg misoprostol was administered rectally, after suturing the episiotomy. 90 min after delivery severe bleeding occurred. The placenta was considered inconspicuous after clinical re-evaluation. Intravenous volume substitution was performed, and ephedrine was administered for circulatory instability, a continuous perfusion of sulprostone was started before confirming the need for surgical revision. The blood loss was estimated at 3500 mL at this point, hemoglobin level was 69 g/L. Consequently, four RBCUs and two units of fresh frozen plasma (FFP) were administered. Curettage was performed but complicated by massive bleeding. Therefore, the patient was transferred to our primary care center. Due to the persistent bleeding and uterine atony, a repeat surgical revision was performed. The cervix showed a small tear, without significant bleeding. Blood clots and placenta-like tissue were evacuated from the uterine cavity. (Eventually, the histopathology revealed only decidua and blood, but no placental tissue.) Bakri^®^-Balloon placement was unsuccessful, and four sulprostone soaked compresses were inserted into the uterine cavity. The patient was stabilized by administration of a further seven RBCUs, eight units of FFP, three units of platelet concentrates (PLT), 2 g tranexamic acid (TXA), 4 g fibrinogen, and activated recombinant human factor seven (rhFVIIIa): However, as the bleeding control was not yet satisfactory, TAE was performed by our interventional radiologists (Figure 2) in order to avoid laparotomy as well as hysterectomy. The right uterine artery and parametran branches of the left uterine artery were embolized with Gelfoam^®^. Post-surgery, the patient was taken to the intensive care unit (ICU) for observation for 24 h. Additional four RBCUs and one TC were administered, as well as 2 g TXA. The following day, the intrauterine compresses were removed, and the patient was transferred to our maternity ward. The patient was discharged 10 days postpartum in generally good health.

The patient reported resumed menstruation after three months, but also chronic abdominal pain since the intervention. Three years after the delivery with subsequent TAE, a spontaneous pregnancy occurred, and the patient vaginally delivered a healthy female newborn at 39 weeks of gestation. A postpartum hemorrhage because of uterine atony reoccurred but was rapidly controlled with uterotonics and TXA. The blood loss was 1000 mL and the patient had a normal puerperium. 

## 5. Discussion

Selective transcatheter arterial embolization (TAE) seems to be an effective method of management of PPH in cases where conservative treatment fails. Overall, the efficacy of embolization in our study was 100%, as PPH was successfully treated in all cases, which is in line with previous publications [9,10,11]. All six reported hysterectomies were performed because of the abnormally invasive placenta and had been previously planned. In all other patients, hysterectomy was successfully avoided by TAE. 

Although TAE has proven to be an effective method, the transitory ischemia associated with the intervention raises the important question of long-term consequences related to the procedure, in regards to fertility as well as to further pregnancies.

Our results suggest that the endometrium is not impaired by TAE, as most women reported resumption of regular menstruation. This observation is in line with most previous studies, as only one publication reports menstrual cycle impairment after TAE [22]. Several previous studies performed on patients who underwent embolization because of fibroids suggest impairment of ovarian function after the procedure [14]. However, the two methods differ, as fibroid embolization aims at permanent ischemia of selective distal arteries. Whether TAE performed in postpartum hemorrhage has an impact on ovarian function has not been analyzed in our series. To our knowledge, this has never been evaluated to date, because of the small available cohorts and short follow-up periods [23]. Sonography shortly after TAE showed reperfusion in the uterine arteries in most of the cases in our series. 

Spontaneous pregnancy occurred in 64% of patients desiring subsequent pregnancy in our cohort (*n* = 13 pregnancies). Nevertheless, only four of the pregnancies resulted in live births, which indicates a high miscarriage rate. Notably, seven of the nine miscarriages occurred in the same patient. The infertility rate reached 36.4%, as four patients failed to conceive, which is clearly higher than in the general population. Two of these patients were referred for assisted reproductive technology (ART) without success. At this point, we would like to mention that all data was obtained without adjusting for confounding factors such as age or male factors, which could have contributed to infertility in our series. Other authors also report higher miscarriage rates after embolization for fibroids, whereas most studies involving TAE in an obstetrical setting report miscarriage rates similar to the general population [23,24]. In most existing studies, fertility does not seem to be affected after TAE, however, all existing reports included small series [25,26,27,28,29]. 

The sample size is also the main limitation of our study, which leads to its broadly descriptive nature. A complex statistical analysis is not possible. A further limitation is the heterogeneity of the methods used for TAE, such as different embolizing agents as well as catheter occlusion. Due to the small number of patients, all women with interventional uterine artery occlusion treated in our hospital have been initially included, however, temporary balloon occlusion patients were subsequently excluded from the fertility analysis, because of the different technique. 

The strength of the study is the sufficient follow-up period (up to 10 years), and uniformity of the data, as obstetrical and radiological interventions were performed in the same primary care center.

Of the patients who delivered a live baby in a subsequent pregnancy, 50% had a recurrent postpartum hemorrhage, which is in line with other studies [16] (Table 5). 

All cases were treated conservatively and did not require a hysterectomy. Interestingly, 50% of our patients gave birth before reaching 37 weeks of gestation, whereas the rate of preterm delivery in the general population in our country reaches 11% [30]. One other study also reported a higher incidence of preterm delivery after TAE [31]. 

Fetal weight at birth was within the normal range in all patients. This observation is in line with most publications [17]. Few studies described cases of intrauterine growth restriction after TAE, suggesting placental insufficiency as an underlying cause [32,33]. This hypothesis remains controversial.

**Table 5:** Comparison of similar series in the literature.

Author, Year of Publication	Size of Cohort (*n*)	Study Period (Years)	Efficacy of TAE (%)	Number of Subsequent Deliveries	Mode of Delivery	Reccurence of PPH in Subsequent Pregnancy
Salomon et al., 2003 [13]	28	5	100%	4	NA	100%
Shim et al., 2006 [19]	43	3	86%	6	5 vaginal, 1 CS	NA
Fiori et al., 2009 [21]	56	10	100%	12	12 vaginal	8%
Hardemann et al., 2010 [26]	53	6	100%	13	9 vaginal, 4 CS	18.1%
Poggi et al., 2015 [31]	103	3	NA	17	7 vaginal, 10 CS	58.8%
Radan et al., 2022	28	10	100%	4	1 vaginal, 3 CS	50%

## 6. Conclusions

Our study shows that TAE is an effective method for the treatment of non-controllable PPH. The method remains an alternative to hysterectomy in women who wish to preserve their fertility, yet patients should be informed of its limitations. Despite uterus preservation, our data suggests that fertility still seems to be limited in these patients. Furthermore, first trimester miscarriage, as well as recurrent PPH, seem to be increased. It remains open if this is the consequence of the underlying cause of PPH or the TAE treatment. The limited number of patients underlines the need for international prospective registers and follow-up studies to further evaluate the outcomes of the procedure. In the meantime, both obstetricians and radiologists should counsel patients who desire pregnancy after TAE for PPH accordingly.

Furthermore, the decision to perform TAE should consider logistic factors such as the availability of experienced professionals and the ability to perform the procedure within an acceptable window of time. 

## Figures and Tables

**Table 1:** Embolization methods and site; TAE = transcatheter arterial embolization.

Embolization	
- Therapeutic (*n*, %)	17 (61%)
- Prophylactic (*n*, %)	11 (39%)
TAE site (artery)	
- uterine artery (uni- or bilateral) (*n*, %)	16 (57%)
- common iliac artery (bilateral) (*n*, %)	3 (11%)
- internal iliac artery (bilateral) (*n*, %)	7 (25%)
- aneurism of the left uterine artery (*n*, %)	1 (4%)
- left pudendal artery (*n*, %)	1 (4%)
Method	
- embolic agents (Embozene^®^/BioSphere^®^, Gelfoam^®^/Spongostan^®^, Coils VortX^®^) (*n*, %)	18 (64%)
- Occlusion catheter (*n*, %)	10 (36%)

**Table 2:** Patients’ characteristics and outcomes.

Age in years (median, +/− SD)	34 (+/− 5)
Nulliparous (*n*, %)	11 (39%)
Multipara (*n*, %)	17 (61%)
Previous CS (*n*, %)	11 (39%)
Previous curettage (*n*, %)	12 (43%)
Previous PPH (*n*, %)	6 (21%)
Uterine fibromas (*n*, %)	2 (7%)
Previous preeclampsia/eclampsia/HELLP	5 (18%)
Congenital bleeding disorder (*n*, %)	2 (7%)
Acquired coagulopathy (*n*, %)	1 (4%)
Histologically confirmed abnormal placental implantation (increta/accreta/percreta) (*n*, %)	13 (46%)
Placenta previa (*n*, %)	10 (38%)
Intake of low dose aspirin (*n*, %)	3 (11%)
Vaginal delivery (*n*, %)	6 (21%)
Vaginal-operative delivery (*n*, %)	4 (14%)
Cesarean section (*n*, %)	18 (64%)
Gestational age at birth, weeks (median, +/− SD)	36.4 (4.42)
Secondary PPH (*n*, %)	1 (4%)
Blood loss (mL, median, +/− SD)	3446 (2749)
Placental abruption (*n*, %)	1 (4%)
Prolonged labour (*n*, %)	3 (11%)
Uterine rupture (*n*, %)	1 (4%)

CS = cesarean section, PPH = postpartum hemorrhage, HELLP = hemolysis, elevated liver enzymes, low platelets, SD = standard deviation.

**Table 3:** Interventions for postpartum hemorrhage; CS = cesarean section.

Conservative Intervention	
Tranexamic acid (*n*, %)	10 (36%)
Uterotonics (carbetocin, oxytoxin, sulprostone) (*n*, %)	14 (50%)
Cell saver (*n*, %)	3 (11%)
Blood transfusion (*n*, %)	22 (78%)
Fresh frozen plasma (*n*, %)	16 (57%)
Platelet transfusion (*n*, %)	8 (29%)
Fibrinogen (*n*, %)	7 (25%)
Activated recombinant human factor seven (rhFVIIIa) (*n*, %)	4 (14%)
Bakri^®^-Balloon (*n*, %)	7 (25%)
**Surgical Intervention**	
Cervix suture (*n*, %)	4 (14%)
Compression sutures (*n*, %)	3 (11%)
Vessel ligatures (*n*, %)	2 (7%)
Curettage (*n*, %)	10 (36%)
Manual placenta delivery (*n*, %)	1 (4%)
Hysterectomy (*n*, %)	6 (21%)
Suture uterus rupture (*n*, %)	1 (4%)

**Table 4:** Fetal outcomes in subsequent pregnancies after TAE.

Gender	Week of Gestation at Birth	Delivery Mode	Birth Weight (g)	Arterial pH	Venous pH	5-Min APGAR Score
male	39 + 6	Vaginal	3345	7.38	7.39	9
female	36 + 2	I° CS	2620	7.33	7.42	9
female	40 + 3	I° Repeat-CS	3095	7.18	7.32	10
female	36 + 1	I° Repeat-CS	2710	7.30	7.35	6

## Data Availability

De-identified data available upon request at ‘anda-petronela.radan@insel.ch’.

## Abbreviations

ART	assisted reproductive technology
CS	cesarean section
FFP	fresh frozen plasma
ICU	intensive care unit
PLT	platelets
PPH	postpartum hemorrhage
RBCU	red blood cell unit
rhFVIIIa	recombinant human factor seven
SD	standard deviation
TAE	transcatheter arterial embolization
TXA	tranexamic acid
